# RESISTANCE TRAINING AND COGNITIVE FUNCTION IN VASCULAR COGNITIVE IMPAIRMENT: A 12-MONTH RANDOMIZED TRIAL

**DOI:** 10.1093/geroni/igae098.1461

**Published:** 2024-12-31

**Authors:** Teresa Liu-Ambrose, Ryan Falck, Elizabeth Dao, Roger Tam, Ging-Yuek Hsiung, Cindy Barha, Walid Alkeridy

**Affiliations:** University of British Columbia, University of British Columbia, British Columbia, Canada; University of British Columbia, Vancouver, British Columbia, Canada; University of British Columbia, Vancouver, British Columbia, Canada; University of British Columbia, Vancouver, British Columbia, Canada; University of British Columbia, Vancouver, British Columbia, Canada; University of Calgary, Calgary, Alberta, Canada; King Saud University, Riyadh, Ar Riyad, Saudi Arabia

## Abstract

**Background:**

Subcortical ischaemic vascular cognitive impairment (SIVCI) is considered the most treatable form of cognitive impairment, due to its modifiable risk factors such as hypertension and diabetes mellitus. Despite the benefits of resistance training (RT) on cognitive function and cardiometabolic health, no study has examined the effect of RT on cognitive outcomes in SIVCI.

**Methods:**

A 12-month single-blinded, randomized controlled trial with 91 older adults with SIVCI who were randomized to: 1) 2x/week progressive RT; or 2) 2x/week balance and tone exercises (BAT; control). Study eligibility included: 1) age 55 years and older; 2) neuroimaging evidence of cerebral small vessel disease; 3) mild cognitive impairment; and 4) the absence of dementia. Measurements occurred at baseline, 6, and 12 months. The primary outcome was the Alzheimer’s Disease Assessment Scale-Cognitive-Plus (ADAS-Cog 13 with additional cognitive tests). Sex differences in RT efficacy was explored.

**Results:**

45 were allocated to the RT group and 46 to the BAT group. COVD-19 impacted the training of 16 participants (9 RT, 7 BAT). After adjusting for covariates and baseline value, participants in the RT group had significantly better ADAS-Cog Plus performance at 12 months (estimated mean difference [RT – BAT]:-0.18; 95% CI:[-0.35, -0.01]; p=0.047). In the sex-stratified analysis, there was a significant effect of the RT on ADAS-Cog Plus performance (estimated mean difference: -0.25; 95% CI:[-0.47, -0.03]; p= 0.028) for females, but not for males.

**Conclusion:**

Our results suggest progressive RT should be considered in the management and treatment of individuals with SIVCI, particularly for females.

